# The Cerebellar Cognitive Affective Syndrome—a Meta-analysis

**DOI:** 10.1007/s12311-019-01060-2

**Published:** 2019-08-07

**Authors:** Narjes Ahmadian, Kirsten van Baarsen, Martine van Zandvoort, Pierre A. Robe

**Affiliations:** 1 Utrecht University Medical Centre, 100 Heidelberglaan, 3584 CX Utrecht, The Netherlands; 2 Department of Neurology and Neurosurgery, Utrecht University Medical Centre, 100 Heidelberglaan, 3584 CX Utrecht, The Netherlands; 3 Psychological Laboratory, Helmholtz Institute, Utrecht University Medical Centre, 100 Heidelberglaan, 3584 CX Utrecht, The Netherlands; 4 Department of Neurology and Neurosurgery, Rudolf Magnus Brain Institute, Utrecht University Medical Centre, 100 Heidelberglaan, G03.126, 3584 CX Utrecht, The Netherlands

**Keywords:** Cerebellum, Cerebellar cognitive affective syndrome, CCAS, Neuropsychological tests, Executive function, Visuospatial, Language, Affection

## Abstract

The cerebellar cognitive affective syndrome (CCAS) was first described by Schmahmann and Sherman in 1998. Despite their clear depiction of the syndrome, it is our experience that the CCAS has not yet found solid ground as a disease entity in routine clinical practice. This made us question the dimension of the CCAS in cerebellar patients. We performed a systematic review of the literature according to the PRISMA guidelines, in order to answer the question whether patients with acquired isolated cerebellar lesions perform significantly worse on neuropsychological testing compared to healthy controls. Studies were selected based on the predefined eligibility criteria and quality assessment. The systematic search resulted in ten studies, mainly observational cohorts consecutively including adult patients with isolated cerebellar lesions. Patients were compared to healthy controls, and neuropsychological investigation was done within one year of diagnosis. Meta-analysis of the twelve tests that were done in two or more studies showed that cerebellar patients perform significantly worse on Phonemic Fluency, Semantic Fluency, Stroop Test (naming, reading and interference), Block Design test and WMS-R visual memory. Cerebellar patients have significant and relevant deficits in the visuospatial, language and executive function domain. This meta-analysis therefore emphasizes the importance of the cerebellar cognitive affective syndrome as described by Schmahmann and Sherman.

## Introduction

In 1998, Schmahmann and Sherman described remarkable neuropsychological deficits in 20 patients with isolated cerebellar lesions. They introduced the cerebellar cognitive affective syndrome (CCAS), characterized by disturbances of executive function, impaired visuospatial cognition, personality change and linguistic difficulties and resulting in a general decline in intellectual functioning (1).

Even more than 20 years after the initial description of the syndrome, it is our experience that the CCAS has not yet found solid ground as a disease entity in routine clinical practice. This may have several reasons. First, since its introduction in 1998, the interpretation of the syndrome has been to the discretion of clinicians, as a strict definition and diagnostic model have been lacking. It was not until last year that Hoche et al. developed and published the CCAS/Schmahmann scale (2). This bedside tool aimed to facilitate physicians in diagnosing the syndrome and to lead to uniformity across studies. Second, the current CCAS literature consists of only small series of patients that are frequently biased by patient selection rather than consecutive inclusion (1, 3–12). Some studies include children, thereby not differentiating between the CCAS and postoperative paediatric Cerebellar Mutism Syndrome (ppCMS) (11, 12). Others include patients with cerebellar pathologies extending to the cerebrum, preventing any correlations between cerebellar structure and neuropsychological function (2, 5, 13–15). Third, neuropsychological tests are not routinely performed in every patient with a cerebellar disorder. Hence, subtle neuropsychological deficits may be overlooked and the CCAS is not recognized (16). Families may therefore accept behavioural changes as part of the disease. The CCAS is also rarely described in adults after cerebellar surgery (17). In contrast, the paediatric postoperative Cerebellar Mutism Syndrome (ppCMS) is much better known, probably because of its relatively high incidence and the devastating clinical picture (18). The ppCMS is characterized by mutism or a severe reduction in speech, combined with emotional lability and behavioural changes (19), and therefore seems to be the more severe counterpart of the CCAS.

The existence of the CCAS is an established fact. It has been very well described in individual patients and in case series. However, the dimension of CCAS in patients with isolated cerebellar lesions has never been investigated. Indeed, a low incidence and low severity of the CCAS at the group-level may explain why the CCAS has not found solid ground in routine clinical practice.

Therefore, we systematically reviewed the current literature regarding the following P(I) CO question (20, 21):

Do adult patients with isolated cerebellar lesions [Patients] perform significantly worse on neuropsychological tests reflecting Schmahmann’s syndrome domains [Outcome] compared to healthy controls [Comparison]?

## Methods

This meta-analysis was conducted according to the PRISMA guidelines (4, 20, 21). Studies were identified from Pubmed, Embase and Cochrane databases. The years considered were 1998 to present, and no language restrictions were applied. Only original cohort studies of adults (> 18 years old) with isolated cerebellar pathology were eligible for inclusion. Outcome measures were expected to be heterogenous, and therefore, the type and scoring of neuropsychological tests were not predefined. The search syntax comprised of a combination of synonyms and variables for anatomical location, neuropsychological evaluation and pathology (as per 01 July 2019; Table 1). When full-text articles were not directly available, the authors were contacted. The search was completed by scanning reference lists of included articles. Deduplication was conducted electronically and manually. Title and abstract of all retrieved studies were screened by two authors (NA and KB). Disagreements between the two reviewers were resolved by discussion after reading full text. As a quality assessment, the risk of bias was estimated according to the following criteria:

- Consecutive inclusion of patients, to avoid selection bias
- Inclusion of more than 20 patients, to avoid type II errors
- Neuropsychological testing in more than 75% of included patients, to avoid errors by missing data
- Inclusion of a control group, in order to compare cerebellar patients to a “gold standard” of healthy individuals who are tested under similar circumstances
- Neuropsychological testing within 1 year of diagnosis, to be able to draw conclusions about possible causal relationships rather than associations

**Table 1:** Search syntax for Embase, Pubmed and Cochrane databases

Anatomical location	AND	Neuropsychological functions	AND	Pathology
cerebel* [ti]		cognit* [tiab] OR language [tiab] OR speech [tiab] OR behaviour [tiab] OR behavior [tiab] OR affective [tiab] OR non motor [tiab] OR non-motor [tiab] OR CCAS [tiab] OR memory [tiab] OR verbal [tiab] OR attention [tiab] OR spatial [tiab] OR learn* [tiab] OR metalinguistic [tiab] OR visuospatial [tiab] OR visualization [tiab] OR planning [tiab] OR executive [tiab] OR emotional [tiab]		hemorrhage [tiab] OR haemorrhage [tiab] OR tumor [tiab] OR tumour [tiab] OR stroke [tiab] OR infarct* [tiab] OR bleed* [tiab] OR lesion* [tiab] OR dentate nucleus [tiab] OR nuclei [tiab] OR SCA [tiab] OR PICA [tiab] OR AICA [tiab] OR ataxia* [tiab] OR disorder [tiab] OR deficit* [tiab] OR impairment* [tiab] OR disease [tiab] OR dysfunction* [tiab] OR degeneration [tiab] OR disturbance* [tiab] OR pathology [tiab] OR damage [tiab] OR abnormal* [tiab] OR atroph* [tiab]

Studies were assigned a maximum score of 10 points (2 per criterion; 2 when criterion was met, 1 when unclear and 0 when criterion was definitely not met) and ranked according to this score. Studies with a score of 8 or higher were included in the final analysis (Table 2). Data were extracted from the studies on number of included patients and controls, type of pathology, time interval, type of neuropsychological tests performed and test results (mean and standard deviation were calculated if not stated in the paper). Tests were included in the meta-analysis when mean and standard deviation of both cerebellar patients and controls were given by more than one study. Review manager 5.0 was used for analysis.

**Table 2:** Quality assessment. Studies were accredited a maximum of 2 points per criterium: 2 when criterium was met, 1 when unclear and 0 when criterium was definitely not met

Authors	Consecutive pt enrollment	> 20 pts enrolled	> 75% underwent NPT	Use of control group	Time interval to assessments < 1 year	Total score
Frank, B (2010)	2	2	2	2	2	10
Frank, B (2013)	2	2	2	2	2	10
Hokkanen, L. S (2006)	2	2	2	2	2	10
Karaci, R (2008)	2	2	1	2	2	9
Exner, C (2004)	2	0	2	2	2	8
Gottwald, B (2004)	1	2	2	2	1	8
Harrington, D.L (2004)	2	2	2	2	0	8
Mak, M (2016)	1	2	2	2	1	8
Neau, J.P (2000)	2	0	2	2	2	8
Bolcekova (2017)	0	2	2	2	2	8

## Results

**Fig. 1:**
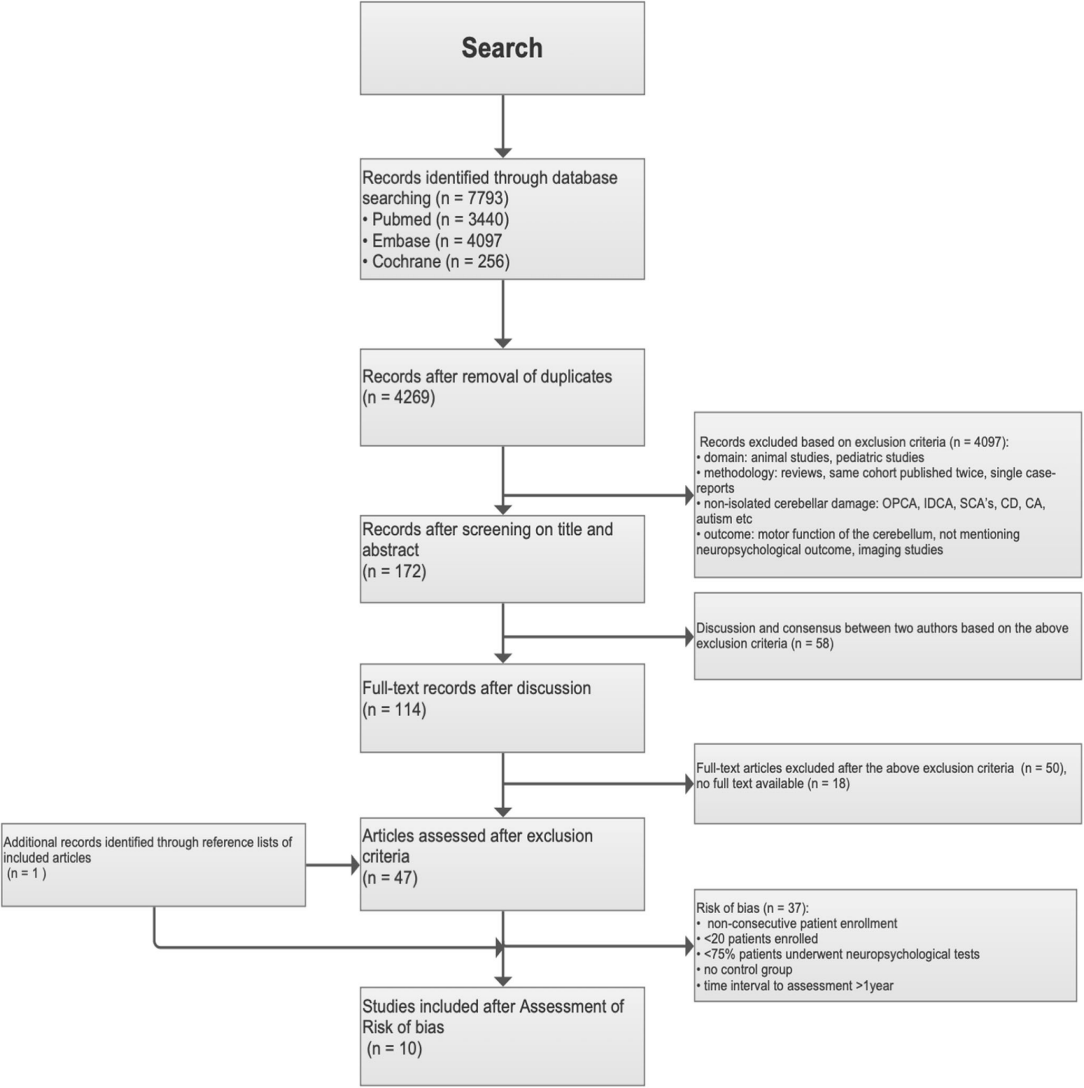
Flow chart of study selection

The search identified 4269 articles from Pubmed, Embase and Cochrane databases, of which 114 were selected based on title and abstract (Fig. 1). Eighteen articles could not be retrieved full-text despite contacting the authors. Only 46 studies fully met the inclusion criteria and after assessment of risk of bias; ten studies were included in the final analysis. The included studies were observational cohorts of patients with isolated cerebellar lesions due to ischemic stroke, tumour or haemorrhage (Table 2). A total of 212 patients, 139 male and 73 females were included in this meta-analysis. Their mean age was 54.0 years (range 18–78) and their years of education 11.7. The control groups were composed of 209 healthy subjects, 132 male and 77 females. They were matched for age (53.6 years, range 18–77) and years of education (11.9), with no neuropsychiatric history, preexisting neurological diseases, past craniocerebral trauma, severe diseases of parenchymatous organs, addiction to drugs and/or alcohol, mental retardation or dementia. Time from diagnosis to neuropsychological assessments was within 1 year in most studies. A minimum of 75% of patients underwent neuropsychological testing.

**Fig. 2:**
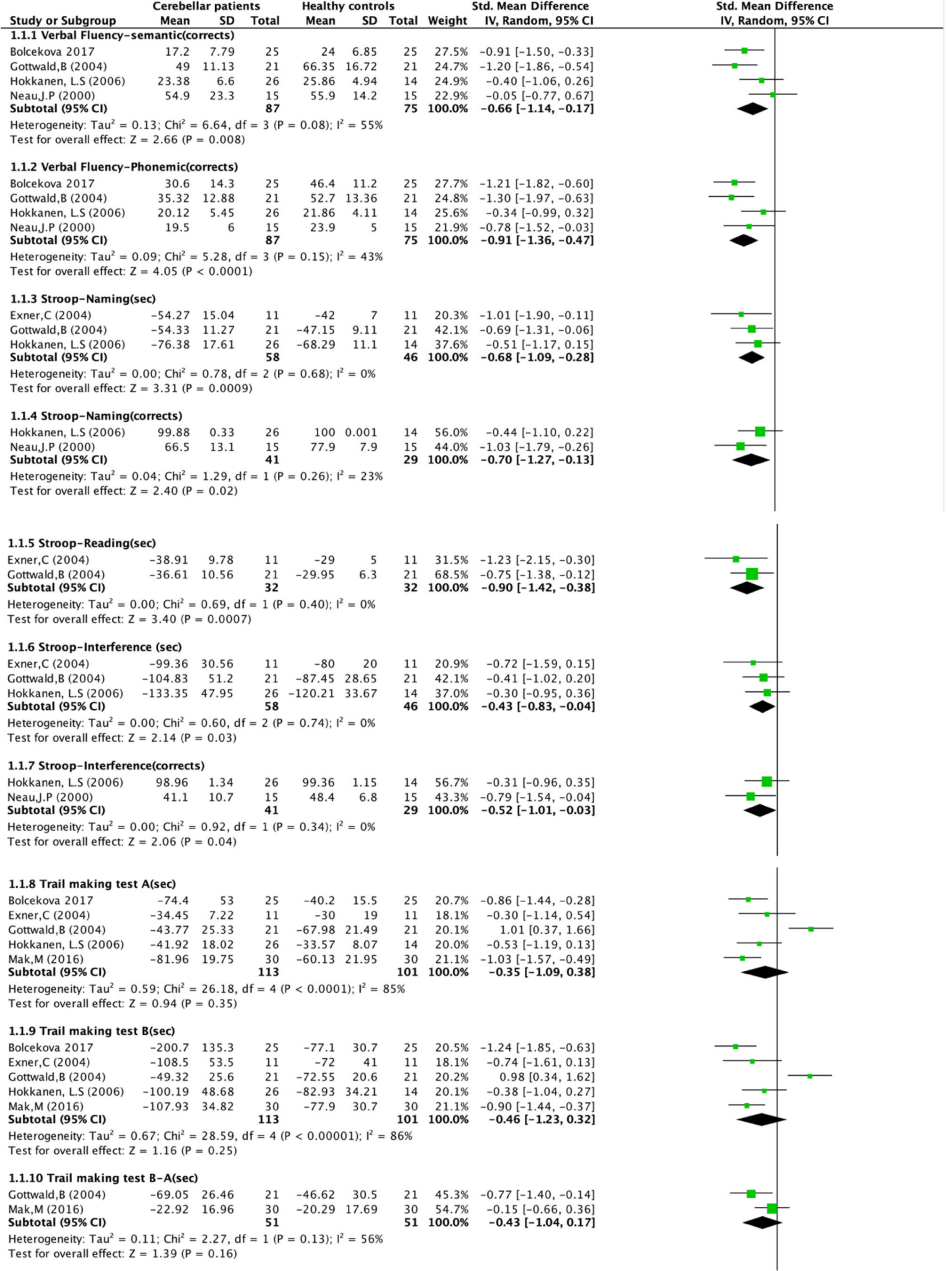

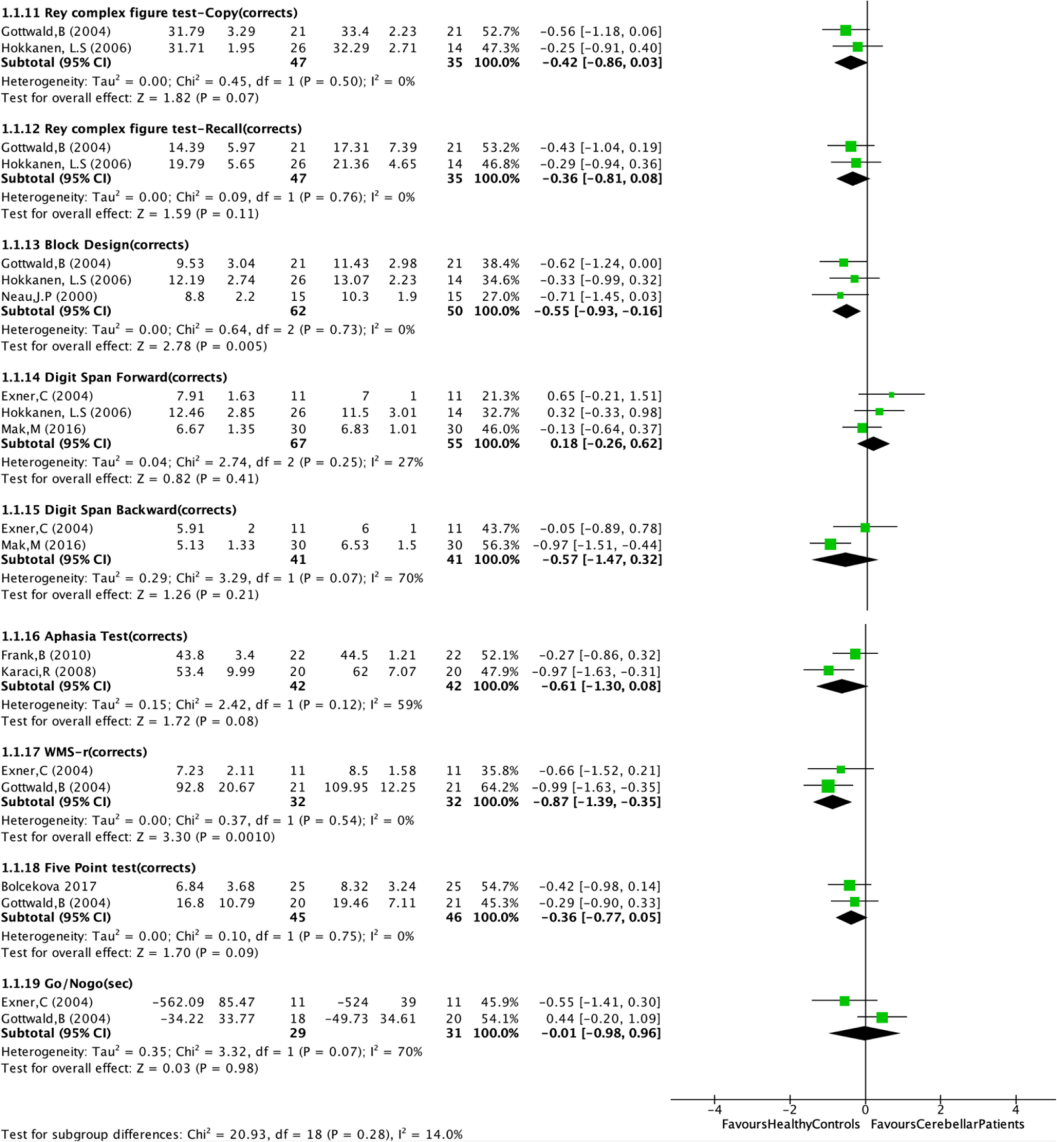
Forest plots of meta-analysis (as per 01 July 2019)

For twelve neuropsychological tests, statistics (mean and standard deviation) were provided in more than one study. These tests could be included in the meta-analysis (Fig. 2): Phonological and Semantic Verbal Fluency, Trail Making Test, Stroop Test, Digit Span Forward and Digit Span Backward, Rey Complex Figure Test, Block Design Test, Aphasia Test, Wechsler Memory Scale- Revisited visual memory test, Five Point Test and Go/No-Go Test (Box 1). Standard mean differences between patients and controls were calculated, considering the different scales that were used in some of the tests.

**Box 1:**
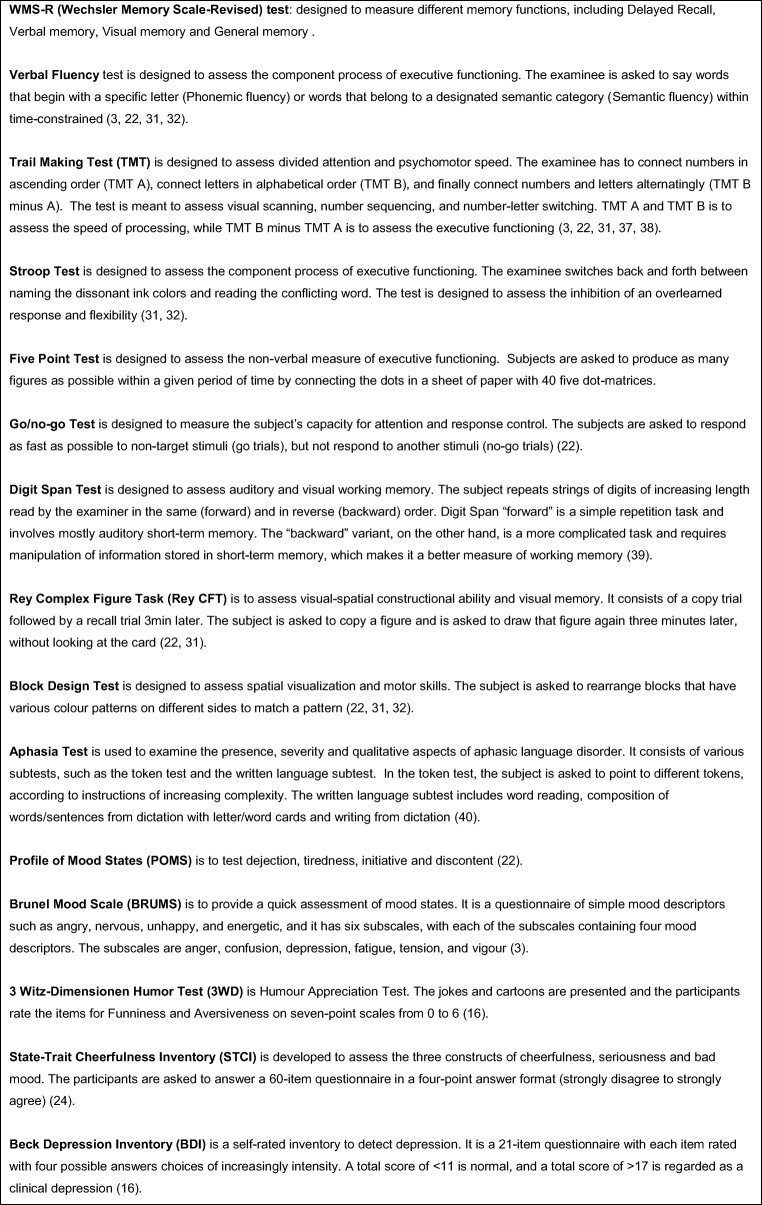
Description of neuropsychological tests

Pooled results in the meta-analysis show that patients with focal cerebellar lesions performed significantly worse in five out of twelve tests (nine out of 19 subtests): Phonological Fluency, Semantic Fluency, Stroop Test (naming, reading and interference), Block Design test (WAIS-R) and WMS-R visual memory. Effect scores varied between 2.06 and 4.05 (Fig. 2).

MS-R (Wechsler Memory Scale-Revised) test: designed to measure different memory functions, including delayed recall, verbal memory, visual memory and general memory.

Verbal Fluency test is designed to assess the component process of executive functioning. The examinee is asked to say words that begin with a specific letter (phonemic fluency) or words that belong to a designated semantic category (semantic fluency) within time-constrained (3, 22–24).

Trail Making Test (TMT) is designed to assess divided attention and psychomotor speed. The examinee has to connect numbers in ascending order (TMT A), connect letters in alphabetical order (TMT B), and finally connect numbers and letters alternatingly (TMT B minus A). The test is meant to assess visual scanning, number sequencing, and number-letter switching. TMT A and TMT B are to assess the speed of processing, while TMT B minus TMT A is to assess the executive functioning (3, 22, 23, 25, 26).

Stroop Test is designed to assess the component process of executive functioning. The examinee switches back and forth between naming the dissonant ink colours and reading the conflicting word. The test is designed to assess the inhibition of an overlearned response and flexibility (23, 24).

Five-Point Test is designed to assess the non-verbal measure of executive functioning. Subjects are asked to produce as many figures as possible within a given period of time by connecting the dots in a sheet of paper with 40 five dot matrices.

Go/no-go Test is designed to measure the subject’s capacity for attention and response control. The subjects are asked to respond as fast as possible to non-target stimuli (go trials) but not respond to other stimuli (no-go trials) (22).

Digit Span Test is designed to assess auditory and visual working memory. The subject repeats strings of digits of increasing length read by the examiner in the same (forward) and in reverse (backward) order. Digit Span “forward” is a simple repetition task and involves mostly auditory short-term memory. The “backward” variant, on the other hand, is a more complicated task and requires manipulation of information stored in short-term memory, which makes it a better measure of working memory (27).

Rey Complex Figure Task (Rey CFT) is to assess visual-spatial constructional ability and visual memory. It consists of a copy trial followed by a recall trial 3 min later. The subject is asked to copy a figure and is asked to draw that figure again 3 min later, without looking at the card (22, 23).

Block Design Test is designed to assess spatial visualization and motor skills. The subject is asked to rearrange blocks that have various colour patterns on different sides to match a pattern (22–24).

Aphasia Test is used to examine the presence, severity and qualitative aspects of aphasic language disorder. It consists of various subtests, such as the token test and the written language subtest. In the token test, the subject is asked to point to different tokens, according to instructions of increasing complexity. The written language subtest includes word reading, composition of words/sentences from dictation with letter/word cards and writing from dictation (28).

Profile of Mood States (POMS) is to test dejection, tiredness, initiative and discontent (22).

Brunel Mood Scale (BRUMS) is to provide a quick assessment of mood states. It is a questionnaire of simple mood descriptors, such as angry, nervous, unhappy and energetic, and it has six subscales, with each of the subscales containing four mood descriptors. The subscales are anger, confusion, depression, fatigue, tension and vigour (3).

3 Witz-Dimensionen Humour Test (3WD) is Humour Appreciation Test. The jokes and cartoons are presented and the participants rate the items for Funniness and Aversiveness on seven-point scales from 0 to 6 (16).

State-Trait Cheerfulness Inventory (STCI) is developed to assess the three constructs of cheerfulness, seriousness and bad mood. The participants are asked to answer a 60-item questionnaire in a four-point answer format (strongly disagree to strongly agree) (29).

Beck Depression Inventory (BDI) is a self-rated inventory to detect depression. It is a 21-item questionnaire with each item rated with four possible answers choices of increasingly intensity. A total score of < 11 is normal, and a total score of > 17 is regarded as a clinical depression (16).

For the remaining tests, the pooled results were also worse in cerebellar patients as compared to controls, although not statistically significant. The only test in which cerebellar patients were not worse compared to healthy controls was the Digit Span Forward.

The affective/emotional domain was addressed by four studies; however, data could not be pooled due to the heterogeneity in tests across these studies. In all studies, overall outcome in the affective/emotional domain appeared not statistically significant. The Profile of Mood Status (POMS), Brunel Mood Scale (BRUMS) and State-Trait-Cheerfulness Inventory (STCI) could not detect overall differences between cerebellar patients and controls (29, 30). Humour comprehension and appreciation and the expression of laughter were not affected as assessed by the 3D Humour Test (16).

Some subtests of these tests, however, did actually show significant differences and always to the disadvantage of cerebellar patients. The Profile of Mood Status (POMS) reached significance for tiredness (22), the Brunel Mood Scale (BRUMS) for confusion, the State-Trait-Cheerfulness Inventory (STCI) detected differences for bad mood and the Beck Depression Inventory (DBI) demonstrated more frequently depressed feelings in cerebellar patients (16).

## Discussion

This meta-analysis shows that cerebellar patients indeed have significant and relevant cognitive deficits.

### Affected Domains

Compared to controls, cerebellar patients perform significantly worse when it comes to processing speed (Stroop reading and naming), executive functions (Stroop interference, Phonemic and Semantic Fluency), memory (WMS-R, visual), language (Verbal Fluency) and visuospatial functions (Block Design test). Effect scores between 2.06 and 4.05 implicate that the differences are not only statistically significant but also clinically relevant. The meta-analysis demonstrates serious deficits in at least three domains: language, executive function and visuospatial. When certain tests did not reach statistical significance after pooling of the data, a trend was always seen towards a poorer performance in cerebellar patients. Pooled data are lacking for the affective/emotional domain, but subtests in individual papers do demonstrate significantly poorer performance in cerebellar patients. The only test in which cerebellar patients did not perform worse compared to healthy controls was the Digit Span Forward test. This implicates that attention and motivation are similar in cerebellar patients as compared to controls. The poorer neurocognitive performance in cerebellar patients can thus not be attributed to a general lack of attention and motivation.

### Correspondence with CCAS/ Schmahmann’s Syndrome Scale

The results from this study therefore demonstrate the significant occurrence of the cerebellar cognitive affective syndrome, as described by Schmahmann and Sherman in 1998, in groups of patients with isolated cerebellar lesions. They also very well match the results from the recently published observational cohort study by the same group (Hoche et al. 2018), which culminated in the diagnostic Schmahmann’s syndrome scale (2). Their study was not eligible for inclusion in our meta-analysis because the study results were not displayed for patients with isolated cerebellar pathology separately. This was a predetermined requirement for inclusion in our meta-analysis in order to correlate cerebellar structure to cognitive function.

Out of the 36 neuropsychological tests that were administered in the observational cohort study by Hoche et al., cerebellar patients performed significantly worse in the vast majority of tests. Based on their differentiating ability and clinical applicability, tests were selected to form the Schmahmann’s syndrome diagnostic scale. Among these were phonemic and semantic verbal fluency and Block design test, comparable to the findings of our meta-analysis. Unfortunately, Hoche et al. did not perform the Stroop test, which according to our meta-analysis would have been a first-choice test for executive function.

### Cerebello-cerebral Diaschisis

The results of this study support a modulatory role for the cerebellum in cognitive functions. A generally accepted hypothesis for the CCAS is that damage to the proximal efferent cerebellar pathways (dentate nucleus, superior cerebellar peduncles) leads to a cerebello-cerebral diaschisis and a hypofunction of supratentorial cortical areas (1, 31). This reversed diaschisis has been demonstrated in single-photon emission computed tomography (SPECT) and perfusion MRI studies. Lower activity in non-motor cerebral cortical areas was shown in patients with the postoperative paediatric cerebellar mutism syndrome (ppCMS), which may be regarded as the paediatric and more severe counterpart of CCAS (29, 32). Functional MRI has further shown a cerebellar representation of the frontoparietal and default mode networks, in addition to activations for cognitive tasks in distinct cerebellar regions (9, 33, 34). This is consistent with findings from virus tracing studies in animals, which have demonstrated that the ventral part of the dentate nucleus is connected with non-motor frontal, temporal and parietal cortical regions (4, 30, 35, 36). All parts of cerebral cortex that receive input through efferents from the dentate nucleus have been shown to send afferents back to the cerebellar cortex via the pontine nuclei, thereby closing the cerebello-cerebral circuitry (4, 9, 30, 35, 36).

Recent functional MRI studies have further confirmed a cerebellar representation of non-motor functions and functional connectivity with cerebral cortical non-motor areas (37–39).

### Level of Evidence and Limitations

Despite the systematic approach and the strict selection of papers, this study offers a level 3a evidence according to the Oxford Centre for Evidence-Based Medicine. The minus refers to the large heterogeneity of the studies included in the meta-analysis. This is largely due to the fact that test procedures were not described in detail, lacking information on test duration and measure units. This explains the large variability in test results (for example, Stroop scores of 98.96 in Hokkanen et al. versus 41.1 in Neau et al. may implicate a longer test duration or even repeated testing by Hokkanen et al.) (23, 24). This large variability in test results culminates in the obvious heterogeneity and may have influenced the results of our meta-analysis. Further, this is a small meta-analysis including few studies, and although common in Medicine, results should always be interpreted with caution (40).

One might argue that there is insufficient evidence for the language domain being affected, as phonemic and semantic fluency tasks reflect executive functioning and semantic memory as well as language (41). Although this meta-analysis does indeed only include phonemic and semantic fluency, there is some evidence for language involvement provided by several other studies that were not included in our meta-analysis due to lower quality (2, 42, 43).

### Clinical Importance

Recognizing the CCAS is of paramount importance for education of patients and their families. It may also initiate dedicated rehabilitation programs focusing not only on executive and visuospatial functions and language but also on psychological well-being and social behaviour. Dedicated treatment programs may lead to better quality of life after cerebellar injury. It is therefore important that the CCAS/Schmahmann’s syndrome scale is actually used and that research is continued in this patient population. Future research should aim for a large prospective observational cohort study including patients with isolated cerebellar injury. The incidence of CCAS in this population should be calculated based on Schmahmann’s diagnostic bedside tool and stratified according to cerebellar pathology. Ideally, studies should include modern imaging techniques focusing on cortical perfusion and cortical function in order to further unravel the pathophysiology of this syndrome.
